# Adverse reactions to antiretroviral therapy: a prevalent concern

**DOI:** 10.26633/RPSP.2017.84

**Published:** 2017-06-19

**Authors:** Cristiane Menezes de Pádua, Letícia Penna Braga, Cássia Cristina Pinto Mendicino

**Affiliations:** 1 Department of Social Pharmacy, School of Pharmacy Universidade Federal de Minas Gerais Belo Horizonte, MG Brazil Department of Social Pharmacy, School of Pharmacy, Universidade Federal de Minas Gerais, Belo Horizonte, MG, Brazil

**Keywords:** Drug-related side effects and adverse reactions, acquired immunodeficiency syndrome, HIV, anti-HIV agents, patient care, Brazil, Latin America, Efectos colaterales y reacciones adversas relacionados con medicamentos, síndrome de inmunodeficiencia adquirida, VIH; fármacos anti-VIH, atención al paciente; Brasil, América Latina, Efeitos colaterais e reações adversas relacionados a medicamentos, síndrome de imunodeficiência adquirida, HIV, fármacos anti-HIV, assistência ao paciente, Brasil, América Latina

## Abstract

The risk–benefit ratio of antiretroviral therapy (ART) is usually considered favorable due to the urgent need to control the HIV/AIDS epidemic. Current studies have shown that combined ART (two or more drugs, from two different classes) is the most effective, with benefits that go beyond clinical management of the disease playing a crucial role in preventing HIV transmission. Therefore, early identification of HIV infection followed by immediate initiation of ART has been encouraged worldwide. However, the success of this strategy has been threatened by poor engagement of patients in HIV care, which may be related to drug harms. In addition, ART is required for the life course, creating the potential for adverse drug reactions (e.g., lipodystrophies). Therefore, adverse drug reactions are a prevalent concern among people living with HIV/AIDS, even in the current era of early initiation of ART (“early ART”), with most drugs considered much safer than those used in previous eras. Accurate diagnosis, recording, and reporting, followed up with proper management and prevention, and intensive surveillance, of new and known adverse reactions to ART, should be strongly encouraged as part of the care continuum.

## RESUMEN

La relación entre los riesgos y beneficios del tratamiento antirretroviral (TAR) generalmente se considera favorable debido a la urgente necesidad de controlar la epidemia de VIH/SIDA. Los estudios actuales han revelado que la politerapia antirretrovírica (dos o más medicamentos de dos clases diferentes) es la más eficaz porque no solo trata la enfermedad, sino que tiene una función crucial como método preventivo de la transmisión del VIH. Por consiguiente, en todo el mundo se ha promovido el diagnóstico temprano de la infección por el VIH seguido del inicio inmediato del TAR. Sin embargo, la efectividad de esta estrategia ha sido comprometida por la baja retención de pacientes en los servicios de salud, que puede estar asociada a seguridad en el uso de los medicamentos. Además, dado que estos pacientes deben recibir TAR durante toda la vida, aumenta la probabilidad de que presenten reacciones adversas a los medicamentos (por ejemplo, lipodistrofias). De esta forma, las reacciones adversas a los antirretrovirales se tornaron una constante preocupación, incluso en la era actual de inicio precoz de la TAR, en que los medicamentos son considerados más seguros que aquellos utilizados en el pasado. El diagnóstico preciso, registro y notificación de las reacciones adversas, seguidos de manejo y prevención adecuados, y vigilancia intensiva para detectar reacciones adversas (nuevas y conocidas) deben ser fuertemente recomendadas como parte de la cascada del cuidado continuado.

## RESUMO

A relação risco-benefício da terapia antirretroviral (TARV) é considerada favorável devido à urgente necessidade de controlar a epidemia de HIV/aids. Estudos recentes demonstram que a TARV combinada (dois ou mais medicamentos de duas classes distintas) é altamente efetiva, apresentando benefícios que ultrapassam o objetivo do manejo clínico da doença, pois contribui de forma significativa para a prevenção da transmissão do HIV. Recomenda-se, portanto, o diagnóstico precoce da infecção pelo HIV e o início imediato da TARV em todo o mundo. Todavia, a efetividade dessa estratégia tem sido comprometida pela baixa retenção de pacientes nos serviços de saúde, que pode estar associada à segurança no uso dos medicamentos. Além disso, o uso da TARV se faz necessário por toda a vida, o que possibilita a ocorrência de reações adversas (como lipodistrofias). Dessa forma, as reações adversas aos antirretrovirais se tornaram uma constante preocupação, mesmo na era atual de início precoce da TARV, em que os medicamentos são considerados mais seguros do que aqueles utilizados no passado. O diagnóstico preciso, o registro e a notificação das reações adversas, acompanhados de tratamento e prevenção adequados, além de intensiva vigilância para detecção de reações adversas (novas e conhecidas) devem ser fortemente recomendadas como parte da cascata do cuidado contínuo.

*Primum non nocere* (“first, do no harm”) is a universal guiding principle in public health that applies to the use of various medical technologies in humans, including drug treatment. In the case of antiretroviral therapy (ART), the risk–benefit ratio tends to be considered favorable given the urgent need to control the HIV/AIDS epidemic (1). Current studies have shown that combined ART (two or more drugs, from two different classes) is the most effective, with benefits that go beyond clinical management of the disease playing a crucial role in preventing HIV transmission (2).

## ANTIRETROVIRAL THERAPY

Mortality and morbidity rates among HIV-infected patients have been considerably reduced since the mid-1990s in both developed and developing countries. This progress is mostly attributable to the use of combined ART regimens containing at least three antiretroviral agents. Currently, six classes of antiretrovirals, comprising more than 25 drugs, are available: 1) nucleoside reverse-transcriptase inhibitor (NRTI), 2) non-nucleoside reverse-transcriptase inhibitor (NNRTI), 3) protease inhibitor (PI), 4) integrase strand transfer inhibitor (INSTI), 5) fusion inhibitor, and 6) C-C chemokine receptor type 5 (CCR5) receptor antagonist (3). However, ART is a lifelong regimen, creating the potential for adverse drug reactions (e.g., lipodystrophies) (4).

Increased knowledge in the field of HIV/AIDS has led to the intensified use of ART to control the disease and prevent HIV transmission. For example, monotherapy with zidovudine (ZDV) in pregnant women has evolved into dual and triple therapy (or therapy with more than three combined drugs) to prevent mother-to-child transmission of HIV. Nowadays, the foci of debate on the recommended treatment are 1) early initiation of ART (“early ART”), regardless of CD4+ cell count and viral load in HIV-infected patients (5–7), and 2) the use of pre-exposure prophylaxis (PrEP) in noninfected individuals at higher risk of HIV infection (8).

## THE HIV CONTINUUM OF CARE MODEL

Current evidence has shown the benefits of early ART initiation in reducing mortality and morbidity of serious AIDS-related diseases (6). Thus, early identification (testing) of HIV infection followed by immediate initiation of ART has been encouraged worldwide for controlling HIV dissemination (5, 6). However, the success of this strategy has been threatened by poor engagement of patients in HIV care (9). The five-stage HIV care continuum or “cascade” model (awareness of HIV infection, linkage to HIV care, retention in care, receipt of ART, and achievement of viral suppression) has been used to guide and measure countries’ progress toward achievement of viral suppression (undetectable viral load). Use of the model entails the description and quantification of people living with HIV/AIDS at each of the five stages of the care continuum trajectory (9). Based on the results of this model, and if the three goals of its “90–90–90” target (90% of patients aware of their diagnosis, 90% on sustained ART, and 90% with undetectable viral load) are achieved by 2020, the Joint United Nations Programme on HIV/AIDS (UNAIDS) has predicted the end of the HIV/AIDS epidemic by 2030 (2).

According to the recently published Global Burden of Disease Study (10), in 2015, an estimated 38.8 million people were living with HIV/AIDS, and there were 2.4 million new infections (corresponding to 1.3 million and 85 000 in Latin America and the Caribbean (LAC) respectively). In 2013, roughly one-third of people living with HIV in LAC countries were unaware of their status (11). Approximately 44% of persons eligible for treatment were on ART, and 34% of them had achieved viral load suppression (11). These estimates were quite heterogeneous across countries, with the worst scenarios found in resource-poor settings with lower coverage of testing and ART (2, 12).

### Adverse reactions to ART and retention in care

Poor adherence to and interruptions of ART among HIV patients receiving care are well-known barriers to the effectiveness of ART (9). Adverse drug reactions are one of the main reasons for interrupting or stopping ART (13, 14). Thus, adverse reactions to antiretroviral drugs might be a hidden threat to the third stage of the HIV care continuum model (retention in care of HIV patients receiving ART). In the current era of “early ART” (2013 to present), the newer, combined antiretroviral regimens are considered safer than those used previously (3). Prior to 2013, HIV patients from LAC countries were on second- or third-line regimens (24% and 5% respectively), with some (about 1%) still receiving obsolete drugs such as didanosine (DDI), stavudine (d4T), indinavir (IDV), and nelfinavir (NFV) (11, 12). These numbers imply that at least 29% of treated patients have already experienced treatment failure and are prone to drug resistance (11), with an estimated prevalence of 7.7% (resistance to any antiretroviral drug) during the 2000–2015 period (15). Considering all these issues, and the need for lifelong treatment (13), people on long-term ART are not expected to be free of risk of experiencing adverse drug reactions at some time.

Adverse effects during the first months of ART are generally highly prevalent (about 60% of treatment-naïve patients (14)), and characterized as mild to moderately severe, and transient, with symptoms that are nonspecific (difficult to distinguish from those occurring due to HIV infection), such as self-limiting neuropsychiatric disorders due to efavirenz (EFV), and gastrointestinal effects due to any antiretroviral drug (14, 16). In comparison, long-term adverse effects tend to be more precise, facilitating the establishment of the relationship between the adverse reaction and the suspected drug (17).

Thymidine NRTIs d4T and ZDV are known culprit drugs of lipoatrophy (fat loss in face, arms, legs, and buttocks) (4). Therefore, recently, in most countries, to reduce the risk of lipoatrophy in HIV patients, d4T was phased out of first-line ART worldwide and ZDV was restricted to alternative or second-line antiretroviral regimens (1). However, until 2013, approximately 53% of patients on treatment in LAC countries were still receiving first-line ART containing ZDV (11). Both PI and, to a lesser extent, NNRTI, widely used antiretroviral classes, are also associated with lipodystrophies (visceral abdominal fat accumulation and fat accumulation of the dorsocervical fat pad, trunk, breast, and neck) (4). Distinct case definitions based on different diagnostic methods have been accepted as the probable explanation for the broad variation in lipodystrophy morbidity (4). Studies have shown a lipodystrophy prevalence of 14% (13) and 7% (17) among patients on prolonged ART.

Frequent metabolic abnormalities (dyslipidemias) and the less common diabetes mellitus type 2 and insulin resistance (well-established cardiovascular risk factors) are another major concern among HIV patients on long-term ART (18). Five-year prevalence of hypertriglyceridemia has been estimated at 40% among Brazilian HIV patients (see (17) for methodological details). Lipid increases have been particularly associated with first-generation PI ART regimens such as those based on IDV and lopinavir (LPV) (total cholesterol, LDL cholesterol, and triglycerides) and EFV (LDL cholesterol) compared to the newer PI regimens (e.g., atazanavir (ATV) and darunavir (DRV)). Diabetes mellitus type 2 and insulin resistance have been found mainly among patients treated with regimens containing first-generation PIs and thymidine NRTIs. All of these metabolic alterations require a more complex drug regimen, which contributes to ART discontinuation or non-adherence among patients on long-term treatment (19).

Low bone mineral density and nephrotoxicity have been reported with the nucleotide analog tenofovir disoproxil fumarate (TDF) contained in most recent recommended first-line antiretroviral regimens for the treatment of HIV/AIDS (16) as well as in PrEP. TDF has been made available together with EFV and lamivudine (3TC, or Epivir in the United States) or emtricitabine (FTC) in a single preparation (“3-in-1” tablet) taken once a day to facilitate adherence. However, the safety profile of TDF should be monitored, with special attention to patients at higher risk of bone diseases and renal impairment (16). Suicide and neurocognitive impairment induced by long-term use of EFV have also been reported, in contrast to the self-limiting neuropsychiatric disorders occurring during the first months of ART (16).

Recently, INSTI-based regimens have been adopted in several guidelines of developed countries as first-line ART. There is some recognition that these drugs have better efficacy and safety profiles than other available antiretroviral classes. The newest INSTI, dolutegravir (DTG), has shown a superior barrier to resistance compared to raltegravir (RAL) and elvitegravir (EVG). Beginning in 2017, DTG will be included in Brazilian guidelines and recommended as a first-line drug (replacing EFV) for both treatment-naïve and experienced patients developing resistance to other antiretroviral drugs. Despite the potential clinical advantages of DTG, some reports of insomnia as well as neuropsychiatric and gastrointestinal adverse events have emerged (20). Therefore, further studies are required to establish the long-term safety of DTG.

## RECOMMENDATIONS

Millions of persons living with HIV/AIDS worldwide (before and since the era of “early ART”) must remain in the care continuum in order to achieve viral suppression. From the perspective of reducing the spread of HIV or providing better patient health care, adherence must be ensured. Therefore, the burden of adverse drug reaction to ART must be monitored as part of the care continuum. Adverse reactions to antiretrovirals remain an issue, even in developed countries, where newer antiretroviral drugs have been made available for use. These reactions will continue to be a prevalent concern among people living with HIV/AIDS on long-term treatment.

Strategies for creating awareness of the risks of ART among HIV patients should be part of HIV programs, including training health professionals to carry out accurate diagnosis, recording, reporting (21), management, and prevention of adverse drug reactions. For example, standardized forms could be used in clinical practice, guiding (compulsory) documentation of key information on adverse reactions associated with ART (17). Availability of high-quality information will be essential for research purposes (17), pharmacovigilance, and generating data for health information systems. The World Health Organization has recognized antiretroviral toxicity surveillance as an integral component of monitoring and evaluation within ART programs (1). According to these guidelines, surveillance approaches (targeted spontaneous reporting, active surveillance within sentinel cohorts, and cohort event monitoring) that best suit the local context should be encouraged and developed to monitor the safety of antiretroviral drugs.

## CONCLUSIONS

Given the effect of ART in control of the HIV/AIDS epidemic, the main barriers to the use of this treatment—adverse drug reactions—must be minimized to ensure the success of this treatment strategy. Therefore, accurate diagnosis, recording, and reporting, followed up with proper management and prevention, and intensive surveillance, of new and known adverse reactions to ART, should be strongly encouraged as part of the HIV care continuum.

## Funding

Part of the data presented in this report is the result of a cohort study sponsored by Brazilian agencies Conselho Nacional de Desenvolvimento Científico e Tecnológico (CNPq) (#474547-2013-2 and #484865/2011-0); Fundação de Amparo à Pesquisa do Estado de Minas Gerais (FAPEMIG) (#APQ0043-10); and Pró-Reitoria de Pesquisa (PRPq) / Universidade Federal de Minas Gerais (UFMG).

## Disclaimer

Authors hold sole responsibility for the views expressed in the manuscript, which may not necessarily reflect the opinion or policy of the RPSP/PAJPH or the Pan American Health Organization (PAHO).
